# A comparison of in vivo and in vitro 31P NMR spectra from human breast tumours: variations in phospholipid metabolism.

**DOI:** 10.1038/bjc.1991.122

**Published:** 1991-04

**Authors:** T. A. Smith, J. Glaholm, M. O. Leach, L. Machin, D. J. Collins, G. S. Payne, V. R. McCready

**Affiliations:** Department of Nuclear Medicine, Royal Marsden Hospital, Sutton, Surrey, UK.

## Abstract

An in vivo 31P NMR spectrum was obtained from each of four human breast tumours. The phosphomonoester and phosphodiester region of each spectrum consisted of a broad peak. Chemical extracts from samples of each of the tumours obtained at resection were examined on a high field strength NMR system. The phosphomonoester region in the spectrum from each extract resolved into three peaks consisting of phosphocholine, phosphoethanolamine and a nucleoside monophosphate. The phosphodiester region resolved into two components, glycerophosphorylcholine and glycerophosphorylethanolamine. Comparing the in vivo and in vitro data from each tumour showed that the contribution of phosphodiester was much lower in the in vitro spectra. We believe this to be a consequence of phospholipid, which would not appear in the aqueous extract, contributing to the phosphodiester peak in vivo.


Br. J. Cancer (1991), 63, 514–516 © Macmillan Press Ltd., 1991

### A comparison of *in vivo* and *in vitro* $^{31}$P NMR spectra from human breast tumours: Variations in phospholipid metabolism

T.A.D. Smith[1], J. Glaholm[2], M.O. Leach[3], L. Machin[4], D.J. Collins[3], G.S. Payne[3] & V.R. McCready[1]

[1]*Department of Nuclear Medicine;* [2]*Department of Radiotherapy;* [3]*Joint Department of Physics;* [4]*Department of Histopathology; The Royal Marsden Hospital and Institute of Cancer Research, Downs Road, Sutton, Surrey SM2 5PT, UK.*

**Summary** An *in vivo* $^{31}$P NMR spectrum was obtained from each of four human breast tumours. The phosphomonoester and phosphodiester region of each spectrum consisted of a broad peak. Chemical extracts from samples of each of the tumours obtained at resection were examined on a high field strength NMR system. The phosphomonoester region in the spectrum from each extract resolved into three peaks consisting of phosphocholine, phosphoethanolamine and a nucleoside monophosphate. The phosphodiester region resolved into two components, glycerophosphorylcholine and glycerophosphorylethanolamine. Comparing the *in vivo* and *in vitro* data from each tumour showed that the contribution of phosphodiester was much lower in the *in vitro* spectra. We believe this to be a consequence of phospholipid, which would not appear in the aqueous extract, contributing to the phosphodiester peak *in vivo*.

The presence of high concentrations of phosphomonoesters (PMEs) in human breast tumours has been demonstrated by several *in vitro* (Degani *et al.*, 1986; Barzilai *et al.*, 1988; Merchant *et al.*, 1988) and *in vivo* (Sijens *et al.*, 1988; Glaholm *et al.*, 1989) studies using $^{31}$P NMR spectroscopy. Sijens *et al.* (1988) reported that the positive response of two breast carcinomas to radiotherapy was accompanied by a decrease in the PME content of the tumours. Further, quantitated changes in PME levels have been observed in a patient undergoing endocrine treatment and subsequently chemotherapy for locally advanced breast carcinoma using a whole body $^{31}$P NMR system Glaholm *et al.* (1989). These findings suggest that the PMEs may be sensitive indicators of tumour cell response to treatment.

*In vitro* studies of chemical extracts from samples of human breast tumours (Merchant *et al.*, 1988) and human cell culture systems (Daly *et al.*, 1987) have suggested that in breast tumours the PMEs consist principally of phosphoethanolamine (PE) with minor contributions from phosphocholine (PC) and several nucleoside monophosphates.

In order to assess the extent to which *in vitro* NMR data are representative of tissue *in vivo*, and to assist in the interpretation of therapy-induced changes in *in vivo* spectra, we are comparing high resolution NMR spectra from extracts of human breast tumours with *in vivo* spectra from the same tumours prior to operation. The present study shows preliminary results from four patients which in three cases are compared with an assessment of necrotic fraction.

*In vivo* measurements were performed using a 1.5 Tesla Siemens Magnetom whole body NMR system. Spectral information specific to the tumour was obtained using a 5 cm surface coil with Conformal ISIS localisation (Sharp & Leach, 1989) (dwell time 0.2 ms; repetition time 2 s). In each case, contiguous slices were taken through the breast to cover the full extent of the tumour. The ISIS voxel was then chosen to measure the whole tumour.

A sample weighing about 0.5 g (<10% of the total tumour) was obtained from each tumour immediately after resection and frozen in liquid nitrogen within 8 min. In the case of tumour 4, we obtained a slice through the tumour and so this sample contained central as well as peripheral tissue. For each of the other three tumours, a piece was sampled from the tumour periphery. After removal of a section for haematoxylin and eosin staining and subsequent determination of necrotic fraction, the samples were shattered and ground to a fine powder in a pestle and mortar under liquid nitrogen. Metabolites were extracted from each sample using a chloroform/methanol/buffer solvent system (Graham *et al.*, 1987). $D_2O$ (final concentration 10%) and methylene diphosphonic acid (2.5 μmoles) were added to the aqueous phase of the extract and the pH was adjusted to 7.4. NMR analysis of the aqueous extracts was carried out on a Bruker Spectrospin AC250 spectrometer operating at 101 MHz (for $^{31}$P). All measurements were performed under proton-decoupled conditions in a 10 mm probe. At least 2,000 acquisitions were obtained from each sample. The parameters used for each acquisition were: sweep width 7937 Hz; acquisition time 0.5 ms; acquisition delay 5 s. (By comparing peak areas from a spectrum acquired using a repetition time of 20 s with those from a spectrum obtained using a 5 s repetition time we have shown that all the spectral components in the aqueous phase of the extracts achieved full relaxation in 5 s). Acquisitions were performed at 20°C. (The extracted sample components were shown to be stable at this temperature for at least 2 weeks). Peak assignments were verified in sample extracts by repeating the NMR mesurement with the addition of commercially obtained metabolites.

Figure 1 shows the *in vivo* and *in vitro* spectra from each tumour. The *in vivo* spectra show prominent PME and phosphodiester (PDE) peaks. Nucleotide triphosphates and diphosphates (NTP and NDP) are present and also inorganic phosphorus (Pi). Phosphocreatine (PCr) is visible in the *in vivo* spectrum from tumours 2 and 4, however in both these cases the selected region was within 0.5 cm of the chest wall, thus contamination due to respiratory motion cannot be excluded.

The *in vitro* spectra from each tumour show that the high energy moieties, NTP and NDP, are less prominent whilst Pi is increased. This may be the result of degradation occurring during the inevitable time delay between the excision and freezing of the tumours. As well as NTP and NDP, the alpha NTP peak contains resonances from nicotinamide adenine dinucleotide (NAD). NAD appears to be particularly stable since in extracts carried out in this laboratory where most of the NTP has disappeared, NAD is still present.

Diphosphodiester (DPDE) is present adjacent to the alpha NTP peak in some of the *in vitro* spectra. In human breast tumours DPDE has been shown to consist primarily of nucleoside diphosphosugars (Merchant *et al.*, 1988).

Correspondence: T.A.D. Smith.
Received 11 July 1990; and in revised form 15 November 1990.

**Figure 1** *In vivo* (upper) and *in vitro* (lower) spectra from four human breast tumours **a**, tumour 1; **b**, tumour 2; **c**, tumour 3; **d**, tumour 4.

Three significant components are observed in the PME region of each of the tumours. We have confirmed that two of the components are PE and PC which are anabolites of the phospholipids, phosphatidylethanolamine and phosphatidylcholine respectively. The areas, relative to total NMR visible phosphorus, of the peaks in both the *in vivo* and *in vitro* spectra from each of the tumours are shown in Table I. For the *in vitro* spectra, peak areas were calculated by peak integration using software available in the spectrometer. A manual simulataneous peak fitting routine, developed in this

**Table I** Areas of peaks, realtive to total NMR visible phosphorus, in *in vivo* spectra (± associated uncertainty estimate (goodness of fit of the Lorentzian model)) (**a**) and *in vitro* spectra (**b**) of four human breast tumours (units: % total phosphorus)

**(a)**

| | Tumour | | | |
|---|---|---|---|---|
| *Peak* | *1* | *2* | *3* | *4* |
| PME | 17 ± 1 | 13 ± 2 | 14 ± 2 | 17 ± 1 |
| Pi | 9 ± 1 | 9 ± 2 | 11 ± 2 | 4 ± 1 |
| PDE | 40 ± 2 | 25 ± 3 | 33 ± 2 | 36 ± 3 |
| PCr | | 9 ± 1 | 5 ± 1 | |
| gamma NTP | 11 ± 1 | 16 ± 3 | 13 ± 1 | 14 ± 2 |
| alpha NTP | 18 ± 2 | 17 ± 3 | 15 ± 1 | 19 ± 2 |
| beta NTP | 5 ± 1 | 12 ± 3 | 9 ± 1 | 11 ± 2 |
| Sample volume ($cm^3$) | 65 | 129 | 89 | 90 |

**(b)**

| | Tumour | | | |
|---|---|---|---|---|
| *Compound* | *1* | *2* | *3* | *4* |
| PE | 10 | 22 | 25 | 36 |
| PC | 8 | 13 | 5 | 3 |
| AMP | 4 | 2 | 10 | 3 |
| Pi | 41 | 37 | 52 | 29 |
| GPE | 4 | – | – | 1 |
| GPC | 13 | 1 | 1 | 1 |
| gamma NTP | 5 | 5 | – | 5 |
| alpha NTP | 13 | 15 | 8 | 16 |
| DPDE | – | 1 | – | 3 |
| beta NTP | 2 | 5 | – | 3 |
| Necrotic fraction | Up to 50% | <10% | None | Not done |

laboratory, was used to estimate the peak areas in the *in vivo* spectra. This provides an associated uncertainty based on the goodness of fit of the Lorentzian model and on spectral signal to noise. This value is included in the table.

The percentage of PME in the *in vivo* spectrum from each tumour is much lower than in the *in vitro* spectrum. Vermeulen *et al.* (1987) showed that in *in vivo* spectra of human brain the T1 relaxation time of the PME peak, which is also known to consist primarily of PE and PC (Pettegrew *et al.*, 1987), is greater than 2 s. It is therefore probable that the much greater relative concentrations of the PME components in the *in vitro* spectra from each tumour is due to signal suppression, which occurs *in vivo* as a consequence of the shorter repetition time used for the *in vivo* measurements. The spectral components in the extracts may also have shorter T1 relaxation times than is the case *in vivo* since the solvent mixture in the aqueous phase contains about 50% methanol.

Although the peak areas, relative to total NMR visible phosphorous, of the PME peak in the *in vivo* spectra show little inter-tumour variation, the ratio of PC to PE varies profoundly between each of the four tumours. The relative concentrations of these two compounds may reflect differences in the relative content of phosphatidylcholine and phosphatidylethanolamine in the membranes of each of these tumours. We are currently investigating this possibility.

The third peak in the PME region of each tumour is a nucleotide monophosphate (NMP). The concentration of NMP and Pi is particularly high in tumour 3 whereas NTP is almost depleted. This suggests that most of the NMP in the *in vitro* spectra has been derived from the hydrolysis of NTP during the short delay between resection and freezing of this tumour. We have shown that PE, PC, GPE and GPC were stable in a human breast tumour left at room temperature for up to 90 min (Smith *et al.*, 1990*a*).

The two resonances in the PDE region consist of glycerophosphorylethanolamine (GPE) and glycerophosphorylcholine (GPC) which are catabolites of phosphatidylethanolamine and -choline respectively. From the table it can be seen that the relative contribution of PDEs in the *in vitro* spectra of tumours 2, 3 and 4 is much lower than in the

*in vivo* spectra. We are currently investigating variations in the concentrations of phosphorus containing metabolites within a tumour and have found that the levels of such metabolites, relative to total phosphorus, are remarkably consistent throughout breast tumours (Smith *et al.*, 1990*b*). Further, since the sample from tumour 4 consists of central as well as peripheral tumour tissue, it is unlikely that the discrepancy in the intensities of the PDE components between the *in vivo* and the *in vitro* spectra, at least in tumour 4, reflects selective tumour sampling. The most likely explanation is that phospholipids contribute to the *in vivo* PDE peak of human breast tumours. Phospholipids have been shown to contribute significantly to the *in vivo* NMR spectra of both liver (Bates *et al.*, 1989; Murphy *et al.*, 1989) and brain (Cerden *et al.*, 1986).

The necrotic fraction in three of the tumours is shown in the table. Tumour 1 is particularly necrotic compared with tumours 2 and 3. Evanochko *et al.* (1984) have suggested that the concentrations of GPE and GPC may reflect the necrotic fraction of a tumour as a consequence of phospholipid degradation. Interestingly the relative concentrations of GPE and GPC in tumour 1, unlike tumours 2 and 3, are very high.

In summary, by comparing *in vitro* $^{31}$P NMR spectra of chemical extracts of human breast carcinomas with *in vivo* NMR measurements from the same patients, the present work has shown: firstly, that the *in vivo* spectra of human breast tumours are not composed primarily of GPE and GPC, but contain dominating resonances from non-water soluble components which are presumably phospholipid in nature. Secondly we have confirmed that the PME peaks consist primarily of PC and PE and that the peak areas, relative to total phosphorous, of PE and PC vary profoundly between each tumour. The relative concentrations of these two compounds may yield useful information about the phospholipid content of breast tumour cell membranes. Further, a large increase in the intracellular concentration of PC (Besterman *et al.*, 1986) has been shown to be one of the earliest responses of tumour cells to growth factors. Thus the ability to distinguish between the components in the PME region of *in vivo* spectra from human breast tumours, currently by *in vitro* extract measurement and in the future by proton decoupled *in vivo* $^{31}$P NMR measurements (Luyten *et al.*, 1989) may be of considerable value in monitoring and predicting the response of tumours *in vivo*.

This work was supported by the Cancer Research Campaign and by the Royal Marsden Hospital.

### References

BARZILAI, A., HOROWITZ, A., GEIER, A. & DAGANI, H. (1988). The phosphate metabolites in benign and malignant tumours-correlation with the content of estrogen and progesterone receptors. *7th meeting of the Soc. Magn. Reson. Med., San Francisco*, p. 631.

BATES, T.E., WILLIAMS, S.R. & GADIAN, D.G. (1989). Phosphodiesters in the liver: the effect of field strength on the $^{31}$P signal. *Magn. Reson. Med.*, **12**, 145.

BESTERMAN, J.M., DURONIO, V. & CUATRECASAS, P. (1986). Rapid formation of diacylglycerol from phosphatidylcholine: a pathway for generation of a second messenger. *Proc. Natl Acad. Sci.*, **83**, 6785.

CERDAN, S., HARIHARA, V., HILBERMAN, J. & 5 others (1986). $^{31}$P NMR detection of mobile dog brain phospholipids. *Magn. Reson. Med.*, **3**, 342.

DALY, P.F., LYON, R.C., FAUSTINO, P.J. & COHEN, J.C. (1987). Phospholipid metabolism in cancer cells monitored by $^{31}$P NMR spectroscopy. *J. Biol. Chem.*, **262**, 14875.

DEGANI, H., HOROWITZ, A. & ITZCHAK, I. (1986). Breast tumours: evaluation with $^{31}$P NMR spectroscopy. *Radiology*, **161**, 53.

EVANOCHKO, W.T., SAKAI, T.T., NG, T.C. & 8 others (1984). NMR study *in vivo* RIF1 tumours: analysis of perchloric acid extract and identification of $^{1}$H, $^{31}$P and $^{13}$C resonances. *Biochim. Biophys. Acta*, **805**, 104.

GLAHOLM, J., LEACH, M.O., COLLINS, D.J. & 5 others (1989). *In vivo* $^{31}$P magnetic spectroscopy for monitoring treatment response in breast cancer. *Lancet*, **i**, 1326.

GRAHAM, R.A., MEYER, R.A., SWERGOLD, B.S. & BROWN, B.R. (1987). Observations of myo-inositol 1,2-(cyclic) phosphate in a Morris hepatoma by $^{31}$P NMR. *J. Biol. Chem.*, **262**, 35.

LUYTEN, P.R., BRUTINK, R.A., SZWERGOLD, B.S. & 5 others (1989). Broadband proton decoupling in human $^{31}$P NMR spectroscopy. *NMR in Bioscience*, **1**, 177.

MERCHANT, T.E., GIERKE, L.W., MENESES, P. & GLONEK, T. (1988). $^{31}$P Magnetic resonance spectroscopic profiles of neoplastic breast tumours. *Cancer Res.*, **48**, 5112.

MURPHY, E.J., RAJAGOPALAN, B., BRINDLE, K.M. & RADDA, G. (1989). Phospholipid bilayer contribution to $^{31}$P NMR spectra *in vivo*. *Magn. Reson. Med.*, **12**, 282.

PETTEGREW, J.W., KOPP, S.J., MINSHEW, N.J. & 4 others (1987). $^{31}$P Nuclear magnetic resonance studies of phosphoglyceride metabolism in developing and degenerating brain: preliminary observations. *J. Neruopath. Exp. Neurol.*, **46**, 419.

SHARP, J.C. & LEACH, M.O. (1989). Conformal NMR spectroscopy: accurate localization to non-cuboidal volumes with optimum SNR. *Magn. Reson. Med.*, **11**, 376.

SIJENS, P.E., WIJRDEMAN, H.K., MOERLAND, M.A., BAKKAR, C.J.G., VERMULEN, J.W.A. & LUYTEN, P.R. (1988). Human breast cancer in-vivo: H-1 and P-31 spectroscopy at 1.5T. *Raidology*, **169**, 615.

SMITH, T.A.D., GLAHOLM, J., LEACH, M.O., MACHIN, L. & MCCREADY, V.R. (1990*a*). A study of the effects of intra-tumour heterogeneity on the $^{31}$P NMR spectrum in human breast tumours. (In preparation).

SMITH, T.A.D., GLAHOLM, J., LEACH, M.O. & 3 others (1990*b*). Phospholipid metabolites and tumour heterogeneity: an *in vivo* and *in vitro* study of human tumours using $^{31}$P NMR spectroscopy. *9th meeting of the Soc. Magn. Reson. Med., New York*, p. 840.

VERMEULEN, J.W.A.H., LUYTEN, P.R. & DEN HOLLANDER, J.A. (1987). Determination of metabolite concentrations from localized $^{31}$P NMRS of the human brain. *6th meeting of the Soc. Magn. Reson. Med., New York*, p. 136.


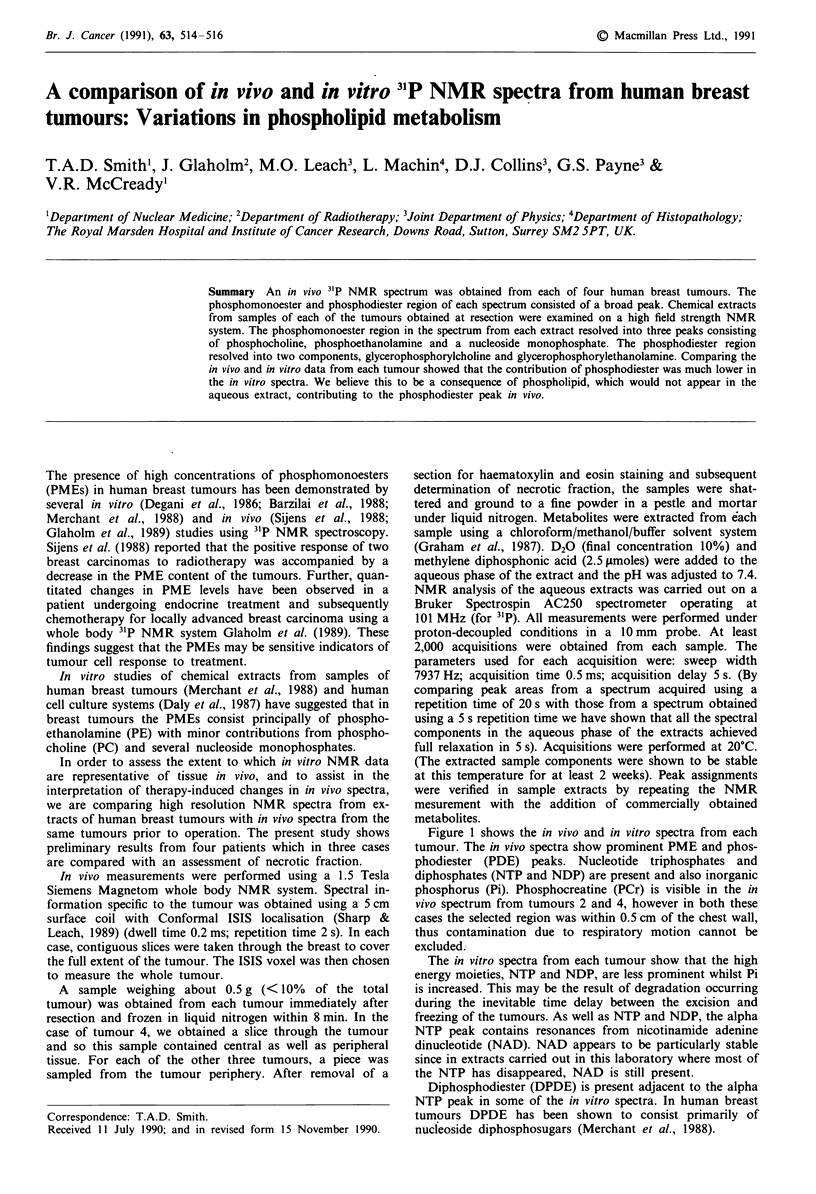

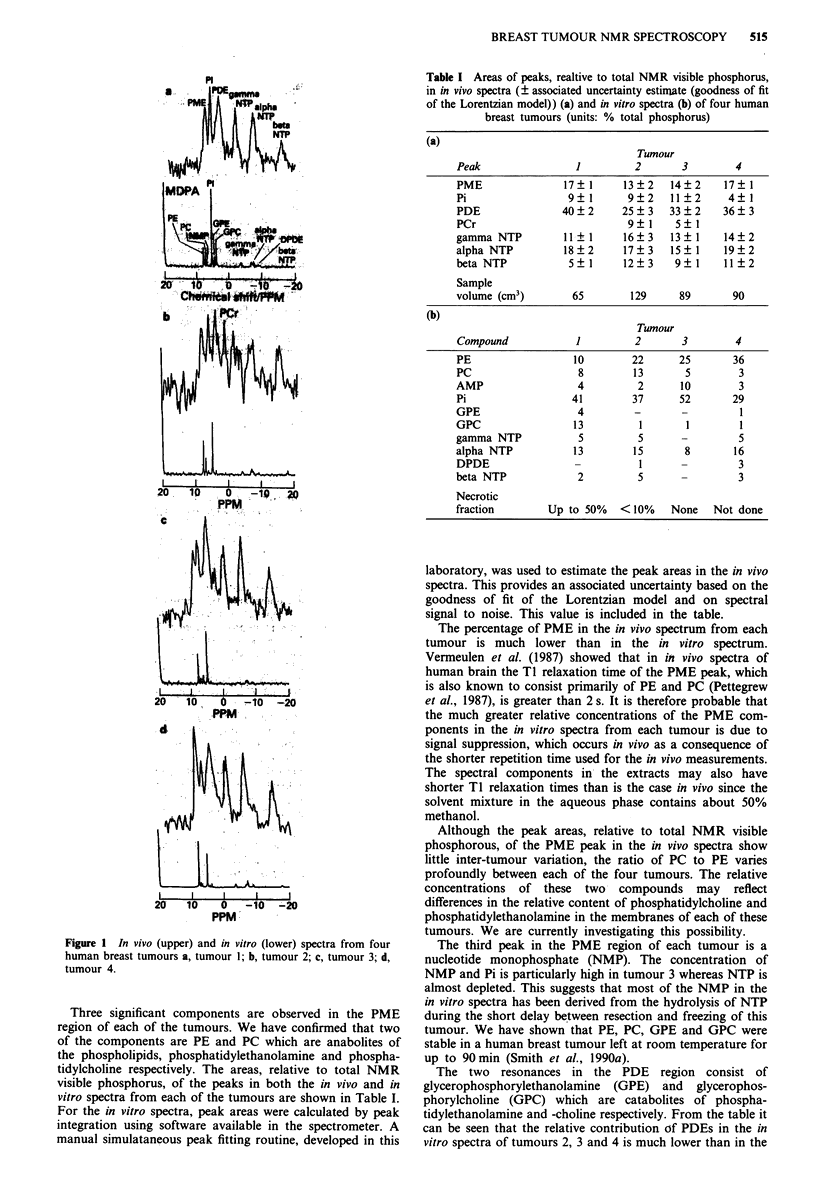

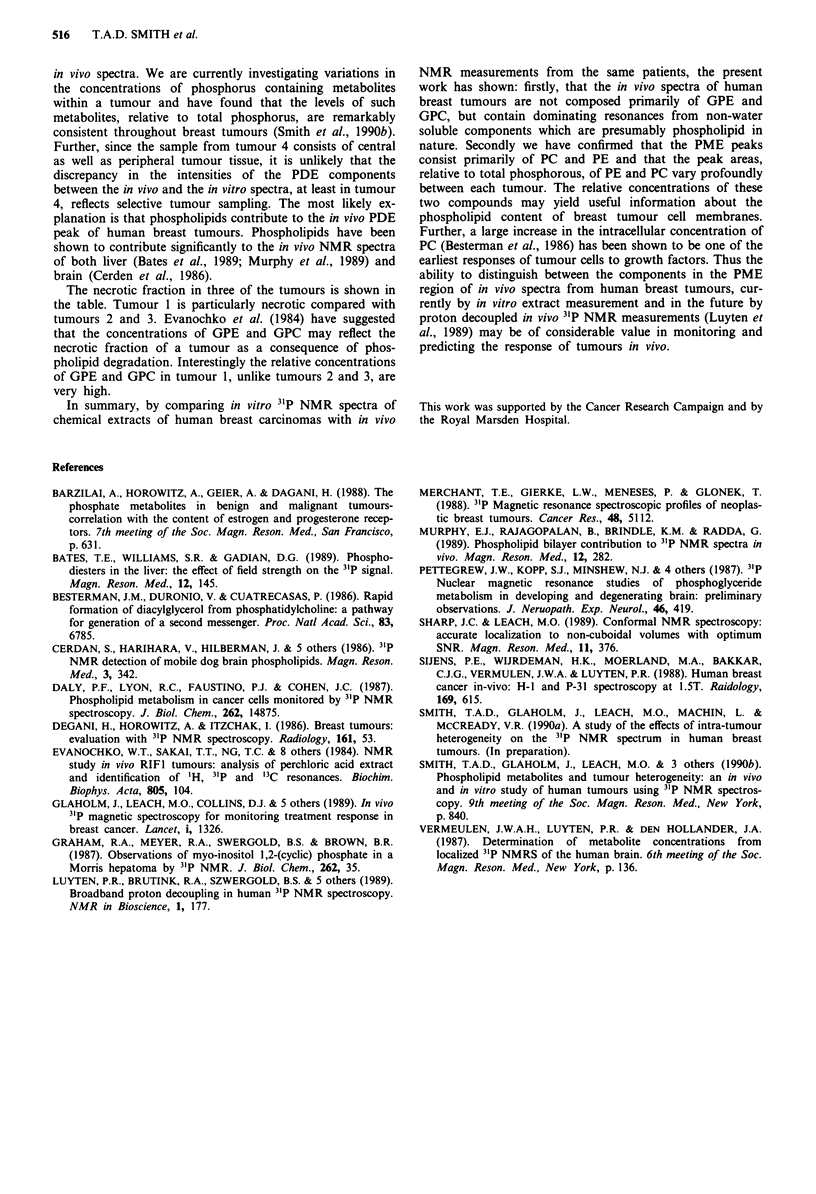


## References

[OCR_00307] Bates T. E., Williams S. R., Gadian D. G. (1989). Phosphodiesters in the liver: the effect of field strength on the 31P signal.. Magn Reson Med.

[OCR_00312] Besterman J. M., Duronio V., Cuatrecasas P. (1986). Rapid formation of diacylglycerol from phosphatidylcholine: a pathway for generation of a second messenger.. Proc Natl Acad Sci U S A.

[OCR_00323] Daly P. F., Lyon R. C., Faustino P. J., Cohen J. S. (1987). Phospholipid metabolism in cancer cells monitored by 31P NMR spectroscopy.. J Biol Chem.

[OCR_00328] Degani H., Horowitz A., Itzchak Y. (1986). Breast tumors: evaluation with P-31 MR spectroscopy.. Radiology.

[OCR_00332] Evanochko W. T., Sakai T. T., Ng T. C., Krishna N. R., Kim H. D., Zeidler R. B., Ghanta V. K., Brockman R. W., Schiffer L. M., Braunschweiger P. G. (1984). NMR study of in vivo RIF-1 tumors. Analysis of perchloric acid extracts and identification of 1H, 31P and 13C resonances.. Biochim Biophys Acta.

[OCR_00338] Glaholm J., Leach M. O., Collins D. J., Mansi J., Sharp J. C., Madden A., Smith I. E., McCready V. R. (1989). In-vivo 31P magnetic resonance spectroscopy for monitoring treatment response in breast cancer.. Lancet.

[OCR_00343] Graham R. A., Meyer R. A., Szwergold B. S., Brown T. R. (1987). Observation of myo-inositol 1,2-(cyclic) phosphate in a Morris hepatoma by 31P NMR.. J Biol Chem.

[OCR_00348] Luyten P. R., Bruntink G., Sloff F. M., Vermeulen J. W., van der Heijden J. I., den Hollander J. A., Heerschap A. (1989). Broadband proton decoupling in human 31P NMR spectroscopy.. NMR Biomed.

[OCR_00353] Merchant T. E., Gierke L. W., Meneses P., Glonek T. (1988). 31P magnetic resonance spectroscopic profiles of neoplastic human breast tissues.. Cancer Res.

[OCR_00358] Murphy E. J., Rajagopalan B., Brindle K. M., Radda G. K. (1989). Phospholipid bilayer contribution to 31P NMR spectra in vivo.. Magn Reson Med.

[OCR_00363] Pettegrew J. W., Kopp S. J., Minshew N. J., Glonek T., Feliksik J. M., Tow J. P., Cohen M. M. (1987). 31P nuclear magnetic resonance studies of phosphoglyceride metabolism in developing and degenerating brain: preliminary observations.. J Neuropathol Exp Neurol.

[OCR_00369] Sharp J. C., Leach M. O. (1989). Conformal NMR spectroscopy: accurate localization to noncuboidal volumes with optimum SNR.. Magn Reson Med.

[OCR_00374] Sijens P. E., Wijrdeman H. K., Moerland M. A., Bakker C. J., Vermeulen J. W., Luyten P. R. (1988). Human breast cancer in vivo: H-1 and P-31 MR spectroscopy at 1.5 T.. Radiology.

